# Structure Characteristics, Biochemical Properties, and Pharmaceutical Applications of Alginate Lyases

**DOI:** 10.3390/md19110628

**Published:** 2021-11-10

**Authors:** Shu-Kun Gao, Rui Yin, Xiao-Chen Wang, Hui-Ning Jiang, Xiao-Xiao Liu, Wei Lv, Yu Ma, Yan-Xia Zhou

**Affiliations:** Marine College, Shandong University, Weihai 264209, China; 201900810266@mail.sdu.edu.cn (S.-K.G.); yinrui@mail.sdu.edu.cn (R.Y.); 202117736@mail.sdu.edu.cn (X.-C.W.); 201900810234@mail.sdu.edu.cn (H.-N.J.); 201900810330@mail.sdu.edu.cn (X.-X.L.); 201900810048@mail.sdu.edu.cn (W.L.); 201900810016@mail.sdu.edu.cn (Y.M.)

**Keywords:** alginate lyases, structure characteristics, biochemical properties, pharmaceutical applications

## Abstract

Alginate, the most abundant polysaccharides of brown algae, consists of various proportions of uronic acid epimers *α*-L-guluronic acid (G) and *β*-D-mannuronic acid (M). Alginate oligosaccharides (AOs), the degradation products of alginates, exhibit excellent bioactivities and a great potential for broad applications in pharmaceutical fields. Alginate lyases can degrade alginate to functional AOs with unsaturated bonds or monosaccharides, which can facilitate the biorefinery of brown algae. On account of the increasing applications of AOs and biorefinery of brown algae, there is a scientific need to explore the important aspects of alginate lyase, such as catalytic mechanism, structure, and property. This review covers fundamental aspects and recent developments in basic information, structural characteristics, the structure–substrate specificity or catalytic efficiency relationship, property, molecular modification, and applications. To meet the needs of biorefinery systems of a broad array of biochemical products, alginate lyases with special properties, such as salt-activated, wide pH adaptation range, and cold adaptation are outlined. Withal, various challenges in alginate lyase research are traced out, and future directions, specifically on the molecular biology part of alginate lyases, are delineated to further widen the horizon of these exceptional alginate lyases.

## 1. Introduction

**Figure 1:**
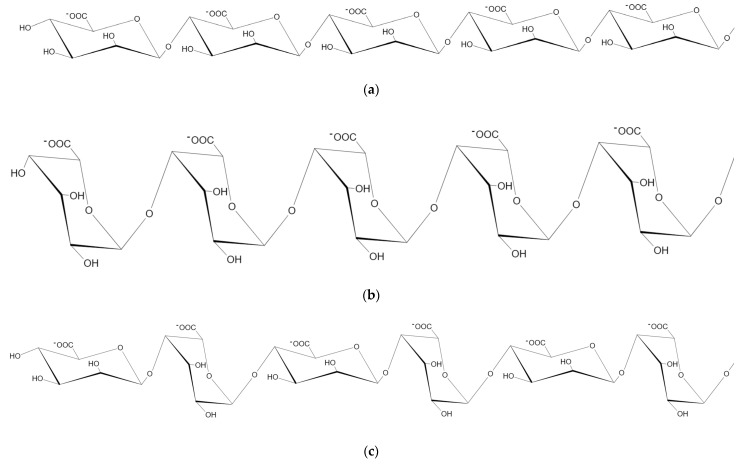
Structure of alginate (M: *β*-D-mannuronic acid; G: *α*-L-guluronic acid). Different blocks are linked by 1→4 glycosidic bonds: (**a**) structure of polyM; (**b**) structure of polyG; (**c**) structure of polyMG.

**Figure 2:**
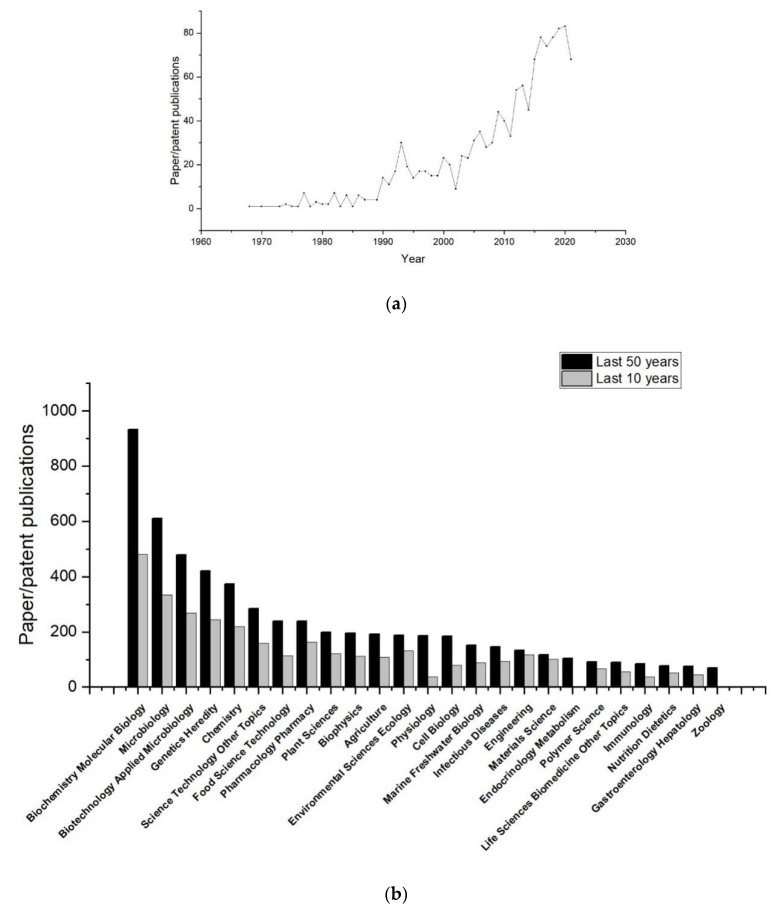
Statistical analysis of published papers and patents about alginate lyases within the past years in Web of science with the key word of “alginate lyase”: (**a**) annual publication of alginate lyases; (**b**) research areas of paper/patent publications of alginate lyases.

Alginate, the structural polysaccharides in brown algae (30–60% dry cell weight) [1], is a linear polysaccharide consisting of two isomer residues, *β*-D-mannuronic acid (M) and *α*-L-guluronic acid (G), which are linked homogeneously or heterogeneously by 1→4 glycosidic bonds to form three different blocks, polyM, polyG, polyMG (Figure 1) [2]. As a high-molecular biopolymer, alginate is most commonly used in the food industry as a thickening agent, since it is limited by its low water solubility and high solution viscosity when high concentrations are required in the pharmaceutical field [3]. Commercial alginates are commonly extracted from brown seaweeds, such as *Macrocystis pyrifera*, *Laminaria hyperborea*, *Laminaria digitata*, *Saccharina japonica*, *Lessonia nigrescens*, *Lessonia trabeculata*, *Ecklonia arborea, Ecklonia radiata*, *Durvillaea potatorum,* and *Ascophyllum nodosum* [4]. Alginate lyases can catalyze the degradation of alginate by *β*-eliminating the glycoside 1, 4-O-glycoside bonds between C-4 and C-5 at the non-reducing end, resulting in the production of oligouronic acids, named unsaturated AOs, or uronic acid monomers. The former can be prepared at high concentrations without a significant increase in viscosity that results in exhibiting excellent bioactivities to impart specific health effects [5]. The latter can be non-enzymatically converted to 4-deoxy-l-erythro-5-hexoseulose uronate (DEH), which can be converted to bioethanol and other chemicals in biorefinery systems [6,7,8,9,10]. As a prerequisite for the production of unsaturated AOs, bioethanol, and biochemical compounds from alginate or brown algae, alginate lyase has received widespread attention, and their related publications keep increasing, especially in the past 10 years (Figure 2a). The statistical analysis from Web of Science (https://www.webofscience.com/wos/alldb/basic-search, accessed on: 13 September 2021) showed that the research reports on alginate lyases have been mainly focused on biochemistry molecular biology, microbiology, biotechnology applied microbiology, genetics heredity, chemistry, and other related fields for 50 years (Figure 2b). Moreover, in the past 10 years, basic research reports on biochemistry molecular biology of alginate lyase are booming, while reports on the pharmaceutical application of alginate lyase are growing rapidly. In view of increasing research on alginate lyase explored recently, a comprehensive understanding of recent progress in alginate lyase is essential for better application. This paper will cover fundamental information, biochemical properties, structure characteristics, and pharmaceutical applications of alginate lyase, in which special properties such as cold adaptation that have been rarely reported, will be generalized. Furthermore, it will show the great potential of alginate lyases and sort out the future direction of their development.

## 2. Alginate Lyases

### 2.1. Sources of Alginate Lyases

Alginate lyases have been derived from many sources including marine algae [11,12], marine and terrestrial bacteria, fungi and viruses, marine mollusks, and echinoderms [1,13]. Marine bacteria account for the largest variety of alginate lyases and are the most widely studied sources of alginate lyases [13]. Interestingly, a novel alginate lyase from human gut microbe *Bacteroides cellulosilyticus* has been recently characterized, which indicated *Bacteroides* from marine or human gut can play a part in alginate utilization [14,15].

### 2.2. Classification of Alginate Lyases

Based on the analysis of primary structure, polysaccharide lyases (PLs) are categorized in 42 families in the Carbohydrate-Active Enzyme (CAZy) database (http://www.cazy.org/, accessed on: 25 September 2021), 14 of which (PL5, PL6, PL7, PL8, PL14, PL15, PL17, PL18, PL31, PL32, PL34, PL36, PL39, and PL41) contain alginate lyases. As indicated in previous studies, alginate lyases from bacteria mostly belong to PL5, 7, 15, and 17 families, while alginate lyases from marine mollusks and viruses generally belong to the PL14 family [16].

According to the substrate specificity [17], alginate lyases can be mainly grouped into three categories: polyM specific lyases (EC 4.2.2.3), polyG specific lyases (EC 4.2.2.11), and bifunctional lyases (EC 4.2.2.-). Researchers may have different understandings of the concept of bifunctional lyases. Some hold that polyMG-specific lyases are not taken into consideration [18] and that polyM-specific or polyG-specific alginate lyases are often confused since they actually have the capacity to degrade other alginates. For example, Aly IV from *Vibrio* sp. QD-5, characterized as polyG-specific lyases, can depolymerize polyM and polyGM with low activity [19]. Moreover, bifunctional lyase does not mean it has the same degradation activity toward both substrates. KJ-2, a bifunctional alginate lyase, preferably degrades the glycosidic bond in M–G linkage than that in G–M Linkage [20]. Another bifunctional alginate lyase, Aly-SJ02, has almost the same activity toward sodium alginate and polyM, while lower activity toward polyG [21], which is in contrast to FsAlgB, whose activity is higher toward polyG [22].

In terms of catalytic modes, alginate lyases can be classified into endolytic and exolytic types. Endolytic alginate lyases cleave glycosidic bonds from the inside chain of alginate polymers and release unsaturated alginate oligosaccharides with different degrees of polymerizations (DPs) as main products, which exolytic alginate lyase can further depolymerize into monomers or dimers [1]. Some exolytic alginate lyases have been reported to directly monomerize alginate to a monosaccharide [23]. Most of the alginate lyases characterized are endotype, and exolytic alginate lyases are rather fewer [24]. Amazingly, alginate lyases with both exolytic and endolytic cleavage activity also exist, such as a novel PL17 family alginate lyase from marine bacterium *Microbulbifer* sp. SH-1 [25].

### 2.3. Catalytic Mechanism

**Figure 3:**
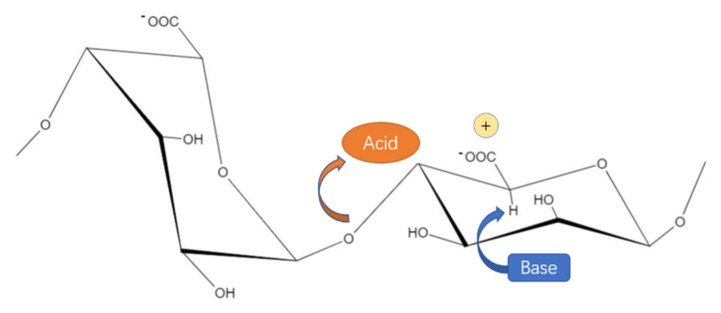
General catalytic mechanism of alginate lyases. Base and acid are specific amino acids in alginate lyases (general Brønsted acid residue: Tyr; general Brønsted base residue: Tyr or His), and “+” refers to positively charged particles (metal ions or amino acids). A Brønsted base residue abstracts the proton on C5 by a general base reaction and a Brønsted acid residue serves as a proton donor.

The action mechanism of alginate lyases is typically *β*-elimination that cleave 4-O-glycosidic bonds between C-4 and C-5 to generate oligosaccharides with 4-deoxy-L-erythro-hex-4-enopyranosyluronic acid at the unsaturated non-reducing end (Figure 3). The reaction can be divided into the following three steps [26]:

(1) Neutralization of carboxyl group on the substrate by a salt bridge;
(2) Abstract the proton on C5 by a general base reaction. The H-5 proton has to be accepted by a Brønsted base residue, and a Brønsted acid residue serves as a proton donor;
(3) Transfer of electrons from the carboxyl group to cleave the 4-O-glycosidic bond, which generates a double bond between C4 and C5. When the 4-O-glycosidic bond is eliminated by the alginate lyases, oligosaccharides containing 4-deoxy-L-erythro-hex-4-enopyranosyluronic acid as the unsaturated non-reducing terminal are created simultaneously, which can be called unsaturated alginate oligosaccharide [1,18,27]. By analysis of configurations at C-4 and C-5, which takes the relative position between abstracted proton and the C-4-bridging oxygen into consideration, the products can be classified as two different configurations: syn-configuration and anti-configuration. For M residues, the C-5 proton and the C-4-bridging oxygen lie syn relative to each other, which is in contrast to G residues, whose C-5 proton and C-4-bridging oxygen lie anti relative to each other [28].

According to differences in the neutralization of the C-5 carboxyl and in the Brønsted bases and acids, the action mechanism can be generally grouped into two different types: His (or Tyr)/Tyr elimination and metal-ion-assisted elimination [28]. In accordance with the preview studies, only a few alginate lyases from the PL6 family adopt the metal-ion mechanism. For instance, a Ca2+ in the active center of AlyGC neutralized the negative charge of the C5 carboxyl group of the substrate [29]. Other alginate lyases belonging to PL5, 7, 14, 15, 17, 18 adopt the His (or Tyr)/Tyr mechanism [30]. The alginate lyases from PL5, 14, and 18 families exhibit the Tyr/Tyr elimination and a Tyr residue is responsible for both the proton acceptor and proton donor [30,31]. The alginate lyases from PL7, 15 families use His residue as the proton acceptor and Tyr residue as the proton donor [32,33,34,35]. As for PL17, most of them exhibit the latter mechanism just as same as PL7 and PL15 [30]. However, one of PL17, Alg17c [36], uses Tyr^450^ as the catalytic base and Tyr^258^ as the catalytic acid. The difference between the two Tyr residues makes it belong to a different mechanism in comparison with PL5, 14, 18. Except for Tyr and His, other residues also play an important role in the activity [37] such as Aly36B from PL36, which replace Tyr residue with Lys residue [30]. Interestingly, a PL6 alginate lyase, AlyF adopts a unique H_2_O-assisted mechanism. H_2_O is demonstrated to stabilize and help orientate the carboxylic group at C5. In addition, Arg293 acts as a catalytic base, and Lys272 acts as a general acid, which cooperates together to finish the further action [38]. The action mechanisms of alginate lyases need to cover special examples, which indicate diverse possibilities in this field.

### 2.4. Structure Characteristics

**Figure 4:**
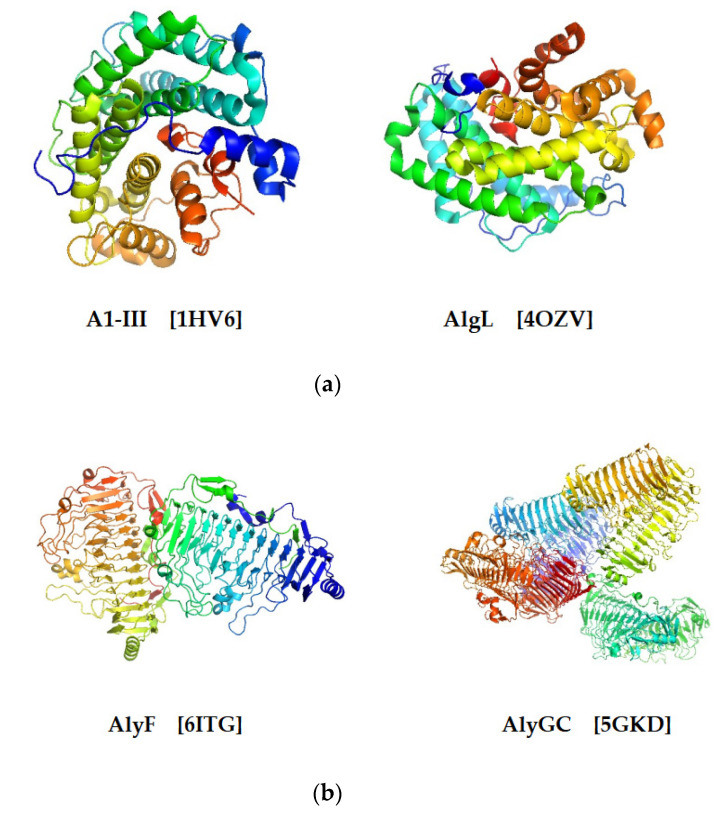

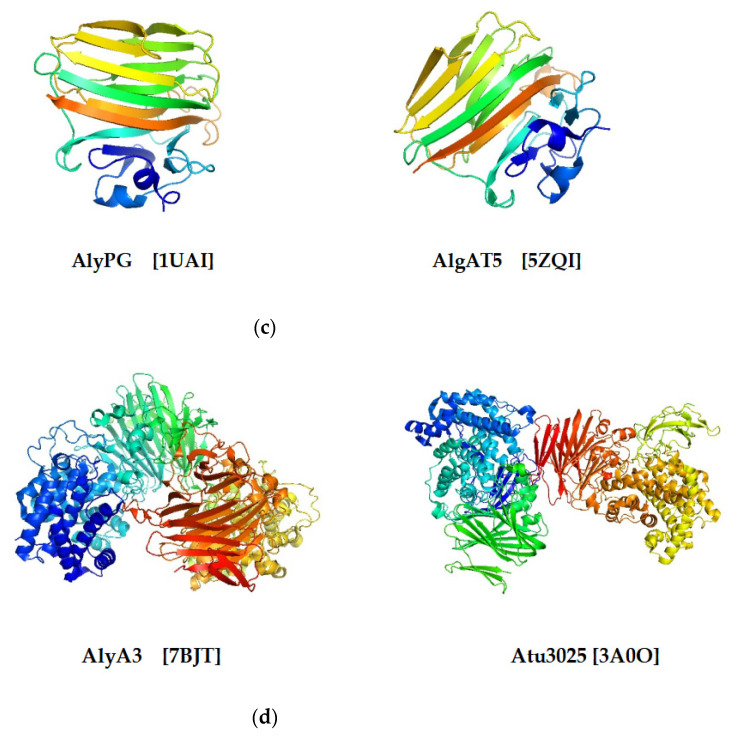
Four different structures of alginate lyases from different PL families: (**a**) alginate lyases adopting (*α*/*α*)_n_ toroid fold structure: A1-III [1HV6], AlgL [4OZV]; (**b**) alginate lyases adopting *β*-helix structure: AlyF [6ITG], AlyGC [5GKD]; (**c**) alginate lyases adopting *β*-jelly roll structure: AlyPG [1UAI], AlgAT5 [5ZQI]; (**d**) alginate lyases adopting (*α*/*α*)_n_ toroid fold + anti-parallel *β*-sheet structure: AlyA3 [7BJT], Atu3025 [3A0O]. All the structures can be found from RCSB PDB (https://www.rcsb.org/, accessed on: 13 September 2021).

With the development of modern structure techniques, more and more structural information on alginate lyases has been revealed in recent years (Table 1). Four kinds of 3D structures are discovered in total as follows:

(1) (*α*/*α*)_n_ toroid fold (Figure 4a). This type of three-dimensional structure is constructed by several antiparallel *α*-helices. The arrangement of helices toroid is counterclockwise looking from the top of it. The majority of alginate lyases from the PL5 family contain only one catalytic domain that adopts a tunnel-like barrel architecture formed by several antiparallel *α*-helices [28]. For example, the active site of A1-III from PL5 is formed by 12 *α*-helices, which form an *α*_6_/*α*_5_ barrel fold with a deep tunnel-like cleft [39]. The structure suggests a mode of action between substrates and catalytic sites, in which substrate molecules penetrate into the tunnel-like cleft and further interact with the catalytic site [39].
(2) *β*-helix (Figure 4b). Alginate lyases from PL6 and PL31 adopt such a structure, which consists of three *β*-sheets (PB) and three turns (T) [28]. The turns (T) are located between two *β*-sheets, respectively, which form a coil of the *β*-helix with PB together: PB1-T1-PB2-T2-PB3-T3 [40]. Several alginate lyases adopting *β*-helix structure have been investigated clearly with the help of structural biology techniques, such as X-ray diffraction. The PL6 alginate lyase BcAlyPL6 is a monomer in solution, and it is comprised of two domains, the N-terminal domain (NTD) and the C-terminal domain (CTD), both of which adopt the right-handed parallel *β*-helix fold [15]. Although the monomer character in solution is different from another PL6 lyase, AlyGC, which forms a homodimer in solution [29], the two domains’ structure is similar to AlyGC and distinct from other structure-determined lyases that adopt this fold but contain only a single domain, such as AlyF [38] and BcelPL6 [14]. Biochemical analysis indicates that the substrate-binding affinity is mainly contributed by the NTD, while CTD of BcAlyPL6 is involved in fixing the substrates into appropriate conformation. However, CTD has weak alginate lyase activity, which may cooperate with the PL6 domain for more effective catalysis. Furthermore, CTD is involved in shaping a closed catalytic pocket, and deletion of it leads to increased activity towards highly polymerized substrate [15].
(3) *β*-jelly roll (Figure 4c). The *β*-jelly roll class, also named as *β*-Sandwich jelly roll, is the most common and most thoroughly investigated fold structure [28]. Up to the time of this publication, four PL families (PL7, 14, 18, 36) adopt such structure, including 12 alginate lyases from PL7, 2 from PL14, 2 from PL18, and 1 from PL36 (Table 1). The structure can be divided into two curved antiparallel *β*-sheets that are linked with each other: the inner concave sheet (SA) and the outer concave sheet (SB) [40]. They further bent in the middle to form a globular shape by nearly 90°. The inner concave sheet plays an indispensable role in catalyzing the reaction, which forms a cleft containing catalytic sites and binding the substrates [28].
(4) (*α*/*α*)_n_ toroid fold + anti-parallel *β*-sheet (Figure 4d). The last fold structure is more complex than the other three kinds of structures mentioned before. This multidomain alginate lyases combine (*α*/*α*)_n_ toroid fold domains with anti-parallel *β*-sheet domains. It is reported that PL15, 17, 39 adopt (*α*/*α*)_n_ toroid fold + anti-parallel *β*-sheet fold structure, including 1 from PL15, 2 from PL17, and 1 from PL39 (Table 1). Ji et al. determined the structure of PL39 whose catalytic domains can be divided into three parts: NTD formed from an incomplete (*α*/*α*)_6_ toroid, central domain constructed by 16 antiparallel strands arranged in two sheets together with a distorted *α*-helix, and CTD consisting of a typical *β*-sandwich [41]. Atu3025 from PL15 adopts *α*/*α* barrel + anti-parallel *β*-sheet fold. Additionally, a pocket-like structure, which is formed by the conformational change at the interface between the central and CTD, has been discovered in Atu3025, which is essential for the exolytic mode of action. This kind of structure is special because it is not reported in other alginate lyases, and lyases that have been reported to have conformational change do not show such pocket-link structure either [35]. The crystal structure of AlyA3 from PL17 has been well investigated [42]. It is similar to another PL17 family lyase, Alg17c, which was the first structure-determined alginate lyase of the PL17 family and organized in two domains, an N-terminal (*α*/*α*)_6_ barrel fused to a C-terminal *β*-sheet domain [36].

The crystal structure of PL8 and PL41 has not been solved yet, which suggests future work should investigate their basic structure to broaden the knowledge about alginate lyases.

### 2.5. Structure–Substrate Specificity Relationship

Recent reviews regarding alginate lyases reviewed the relationship between structure and catalytic efficiency [1,18,28,40,55]. This paper focuses on summarizing the structure–substrate specificity relationship, for substrate specificity is a key factor of the catalytic efficiency and pharmaceutical applications.

**Figure 5:**
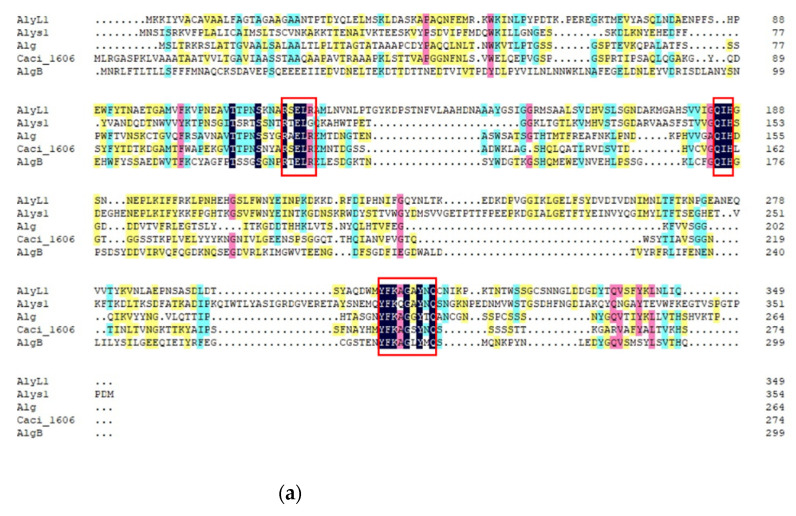

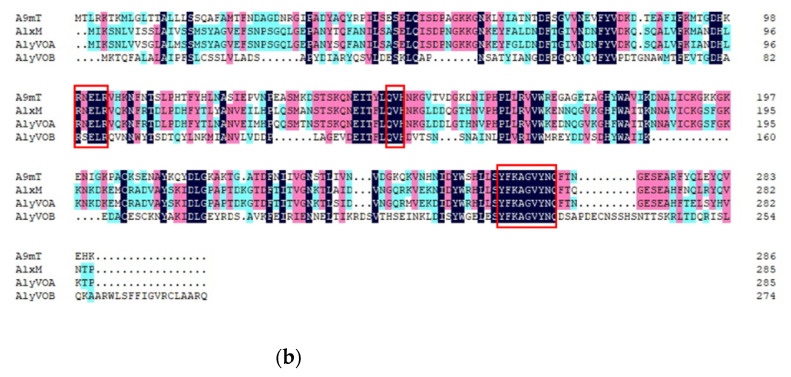
Multiple sequence alignments of PL7 alginate lyases. AlyL1 (AIY68670) from *Agarivorans* sp. L11, Alys1 (OBQ55419) from *Tamlana* sp. s12, Alg (ACN56743) from *Streptomyces* sp. M3 (2009), Caci_1606 (ACU70527) from *Catenulispora acidiphila* DSM 44928, AlgB (ATJ01131) from *Flammeovirga* sp. NJ-04, A9mT (BAH79131) from *Vibrio* sp. A9m, AlxM (CAA49630) from *Photobacterium* sp. ATCC 43367, AlyVOA (ABB36771) from *Vibrio* sp. O2, AlyVOB (ABB36772) from *Vibrio* sp. O2. The conserved amino acid regions are highlighted with red boxes: (**a**) alginate lyases containing QIH in conserved regions; (**b**) alginate lyases containing QVH in conserved regions.

Previous studies focused on the primary structure of alginate lyases to understand the relationship between structure and specific substrate. However, more and more studies have been reported that did not conform to the laws summarized from the perspective of the primary structure. As the largest PL family of alginate lyases, PL7 contains three highly conserved domains: SA3 (RXEXR), SA4 (YXKAGXYXQ), and SA5 (QXH), which have been reported to determine the substrate specificity (Figure 5a,b). Generally, alginate lyases containing QVH in conserved regions show polyM specific activity, while alginate lyases containing QIH in conserved regions show polyG specific activity or polyMG activity [1]. However, many alginate lyases from the PL7 family do not conform to these rules, such as AlyA-OU02, AlyPM, FlAlyA, and AlgSH7 [44,56,57,58]. These opposite examples show that it is incomprehensive to determine substrate specificity only by considering the primary structure. Similarly, sequence-based prediction of PL6 specificities by a Pfam domain search is currently not reliable [14].

Some studies have focused on the three-dimensional structure of alginate lyases. Lid loop, a common structure found in three-dimensional structures, was demonstrated to play an important role in substrate binding and substrate specificity. The structure analysis of AlyF indicated three loops are associated with the long substrates binding, two of which are related to further entry of the substrate into the active region to complete the binding owing to the flexibility of the two loops [59]. The function of loops in A1-II′ have also been demonstrated, and mutation test, which introduces a rigid interaction between the two loops by replacing two residues Asn141 and Asn199, belonging to loop1 and loop2, with Cys, reduced affinity and activity of mutants, which indicate that Asn141 and Asn199 are two key residues in binding the substrates by forming hydrogen bonds, and that flexibility in lid loops is essential for substrate binding [32]. Another PL14 alginate lyase, AkAly30, adopts a special substrate recognition mode, and three key residues in η2 loop are important when considering the substrate specificity: Gly118 and the disulfide bond formed between Cys115 and Cys124 control the conformation of an active-site loop, which makes the space suitable for substrate entry into subsite −1. Both the substitution of Gly118 to an Asn residue on the η2 loop and replacing Cys115 and Cys124 with Ala and Gly resulted in a decrease in substrate specificity toward the polyM. Therefore, together with another η4 loop leading to a closed space of subsite −1 in the AkAly30 structure, η2 regulates the binding mode of the M residue at subsite −1 [52].

Even though the basic roles between substrate specificity and structure are still not clear, research focus has gradually shifted from the primary structure of enzymes to the spatial structure. Additionally, the lid loop of each alginate lyase is a key factor when considering the relationships between the two of them. Moreover, the lid loop is also a critical structure related to other characteristics of alginate lyases, including substrate affinity, catalytic efficiency. For instance, the lid loop of AlgL-CD above the active center presented the transformation progress of “open–close–open” before and after the substrate entered the active site. However, loops of one of the mutants, E226K, created by introducing alkaline amino acid residues near the active center, stayed in a constantly open state, which could make the product leave the active center faster without waiting for transformation progress. Therefore, time and energy could be saved to promote the processivity of the substrate degradation by E226K to enhance catalysis efficiency. Moreover, in view of the negative charge, Lys at the binding site of the non-reducing end of the substrate was more conducive for substrate recognition and binding, resulting in an increase in substrate affinity [60].

### 2.6. Special Properties of Alginate Lyases

There are no reviews on special properties of alginate lyases that help alginate lyases adapt to the living environment and are also conducive to future applications. It is necessary to provide an outline of special enzymatic properties of alginate lyases, such as their cold-adapted, thermostable, high-alkaline, and salt-activated characteristics.

#### 2.6.1. Salt-Activated Property

Alginate lyases isolated from marine environments often possess this property, which helps them adapt to high salinity conditions. With an appropriate concentration of NaCl, their activities can increase by multiples (Table 2).

Although many alginate lyases have been identified to possess this property, the mechanism behind this phenomenon has not been fully revealed. Several research studies reported the possible mechanisms; however, the effects of NaCl were completely different in these alginate lyases. AlgNJ-04 may be activated by it due to the removal of bound water from the sodium alginate molecule or the charge effect in the formation of alginate enzyme complexes [16,65]. However, for AlgM4 [61], the presence of NaCl changes contents of *α*-helix and *β*-sheet in secondary structure (*α*-helix: 12.4%→10.8%; *β*-sheet: 38.2%→36.5%), which may increase the affinity of the enzyme for its substrates and facilitate enzymolysis. As regards AlyPM [58], the test results were similar to AlgM4, which showed a significant increase in the affinity of AlyPM for its substrate when NaCl was added, but CD spectra did not detect any obvious changes between AlyPM in 0 M NaCl and 0.5 M NaCl, which indicated that the enhanced effect of NaCl was not caused by structural changes. Different from them, AlyC3 [47] also has its own mechanism, which is achieved by affecting the aggregation states. As the results demonstrated, the effect of NaCl was embodied in retaining a dimeric quaternary structure of AlyC3. Unfortunately, the research did not report whether the presence of NaCl would affect the affinity of AlyC3 for its substrates. In order to make better use of the optimum conditions of alginate lyases, more studies should be carried out to reveal the mechanism of salt activation.

#### 2.6.2. Wide pH Adaptation Range and Alkaline Property

For most of the alginate lyases, the optimal conditions are close to a neutral pH and only exhibit high activity in a narrow pH range, especially for enzymes from the PL7 family. Additionally, there are some alginate lyases that show the optimal pH in alkaline environments but show activity in a narrow pH range [58,61]. However, as asserted in previous studies, some enzymes exhibit activity in a broad pH range, and they can retain their activity for a long time in incubation tests, which indicated a pH-stable property. For example, Aly08 from PL7 can remain more than 80% of its initial activity in a wide pH range (4.0–10.0), and its optimal pH was found to be 8.35 [64]. In particular, it can retain more than 60% of initial activity in a wide pH range from 7.0 to 11.0 after incubation in different buffers at 4 °C for 12 h. Similar to Aly08, Alyw202 from PL7 exhibited the highest activity at pH 9.0, and it showed a wider pH range than Aly08 [66]. Alginate lyases with excellent pH stability and a wide pH range could be candidates for hydrolyzing acid or alkaline pretreated alginate, which are generally needed to produce alginate oligosaccharides [16]. Here, this paper summarizes the alginate lyases proved to be pH stable (Table 3).

The optimum pH of most alginate lyases with broad pH stability is alkaline, and some alginate lyases even possess optimum pH up to 10 or even higher [63,69]. However, there are some alginate lyases with optimal pH 7.0, such as AlgNJ-04 [65], which can hold more than 80% of its maximum activity at pH 4.0 and 10.0. In addition, high alkaline alginate lyases without a wide pH range stability also exist, such as TsAly6A [70], whose optimal pH is 8.0, but more than 80% of its initial activity after incubation remains at pH ranging only from 6.6 to 8.95 for 12 h. Therefore, even though many alginate lyases exhibit peak activities at a high alkaline pH and adapt to a broad pH range at the same time, there is no absolute correlation between them, which suggests that conclusion should be determined on the basis of experiments, and we cannot simply construct the simultaneous existence of several characteristics.

#### 2.6.3. Thermostable Property

Thermostable or heat-stable property is one of the most important factors in the application of alginate lyases. Owning to high thermostability, the catalytic reaction can proceed at a higher temperature, which can facilitate the transformation of the substrate due to the reduction in viscosity of the reaction mixture and improvement of enzyme activity. Moreover, high temperature can decrease the risk of bacterial contamination when crude substrate such as kelp powder is transformed [71]. Finding alginate lyases possessing high thermostability will be of great importance for both industrial and commercial purposes. It is previously reported that most of the characterized alginate lyases show the maximum activity around 30–40 °C, and their activity decreases as the temperature increases [16,72], while alginate lyases with thermostability generally show a higher optimal temperature than mesophilic homologs and they can also retain a relatively high activity after incubation at a high temperature for more than 30 min (Table 4). One of the most thermostable alginate lyases, AMOR_PL17A from PL17, could magically retain 100% activity after incubating for 24 h at 60 °C in the absence of substrate, and the temperature had a great effect on its activity, as reflected in the product yields, which were 18 folds higher at 90 °C than that at 40 °C [73]. Here, this paper summarizes alginate lyases possessing thermostability that have been published in recent years (Table 4).

Only a few alginate lyases have been revealed to have a mechanism of stability. For example, the heat stability of NitAly was possibly attributed to the disulfide bond forming between two residues—Cys-80 and Cys-232—which was confirmed by 5 mM DTT and mutant experiments [74]. Moreover, the thermal stability of PyAly (another kind of alginate lyase) was magically enhanced by introducing the two Cys residues without decreasing its alginate lyase activity [74]. In addition, the thermostability of AlgC-PL7 was demonstrated to be associated with the contents of *α*-helix [75].

#### 2.6.4. Cold-Adapted Property

As one of the rare properties of alginate lyases, cold adaptation has gradually attracted attention recently for its unique features and potential applications. Here, this paper summarizes the past reports on cold-adapted activity, including its basic characteristics (Table 5) and applications.

##### Basic Characteristics of Cold-Adapted Property

Contrary to thermostable and most reported alginate lyases, cold adaptation means a lower optimal temperature and thermostability. The optimal temperatures for most alginate lyases are not higher than 35 °C, becoming unstable when temperatures increase above 30 °C [70,77]. In addition, more than 50% of their maximum activity generally remains at 20 °C [77]. All the characteristics above make these enzymes possess unique advantages under low temperatures and have the potential to become a powerful tool for industrial applications.

##### Advantages of Cold-Adapted Alginate Lyases

Possessing low optimal temperature and low thermostability, cold-adapted alginate lyases catalyze the enzymatic reactions at low temperature, which can save energy and reduce the risk of microbial contaminations [70,78]. Moreover, such catalytic condition is crucial to improve the sustainability of enzyme utilization [79]. Therefore, making full use of cold-adapted alginate lyases can reduce the cost of industrial production to a certain extent. In addition, due to the poor thermostability, compared with mesophilic homologs, the reaction catalyzed by cold-adapted alginate lyases can be easily and selectively terminated by slightly elevated temperatures, which have advantages in industrial processes, especially those that high temperatures did not allow [81].

##### Cold-Adapted Alginate Lyases Excreting Bacteria

It is worth noting that reports on cold-adapted alginate lyases-excreting bacteria are also rare. Overall, 21 alginate lyases-excreting strains belonging to 5 genera were isolated from 6 Laminaria samples collected from the Arctic Ocean [11], 11 of which could produce alginate lyases with the highest activity at 20–30 °C, indicating good cold-adapted properties. The study on cold-adapted alginate lyases-excreting bacteria from *Laminaria* provided us with a good method to screen the alginate lyases with cold-adapted properties from natural environments and also a good material for associated bacteria learning.

### 2.7. Strategies for Improving Application Ability

Enzyme immobilization and molecular modification are two common methods to improve enzyme activity [85]. Enzymes are attached to support materials to achieve immobilization and according to the different connection modes, the attachment can be divided into covalent bonding, adsorption, entrapment, and cross-linking [18]. Here, this paper mainly focuses on a molecular modification that improves the application ability of alginate lyases at a molecular level.

Except to improve the efficiency described previously [18,40], molecular modification is mainly operated to change the properties of the enzyme for better application. Rational design, semi-rational design, directed evolution, conserved domain recombination, and non-catalytic domain truncation are common strategies leading to many kinds of mutants that possess different properties from the original enzyme [18,40]. More and more examples have revealed the feasibility of molecular design in changing the activities of alginate lyases. Xu et al. [60] removed signal peptide and carbohydrate-binding domain of alginate lyase AlgL from *Pseudoalteromonas* sp. zb-7 and expressed its catalytic domain as a mature alginate lyase AlgL-CD. Furthermore, they achieved a rational design by introducing alkaline amino acid residues near the active center to enhance its activity. Additionally, a remarkable finding was that the activity of one mutant, E226K, was significantly increased, and the substrate affinity of E226K increased by 10 times, compared with the wild-type AlgL-CD. Another typical rational design is the introduction of disulfide bonds, and many research studies have adopted such methods to improve the thermal stability of alginate lyases. For instance, studies used rational design (by introducing disulfide bonds) to enhance the thermal stability of cAlyM from *Microbulbifer* sp. Q7 [86]. Mutants D102C-A300C and G103C-T113C were expressed in *E. coli*, while mutant 102C300C was expressed in *P. pastoris.* They all have better thermal stability than wild-type cAlyM, but alginate lyases expressed in *P. pastoris* are safer and more convenient than those expressed in *E. coli* [87]. It is also indispensable to observe that H-bonds changed in D102C-A300C, and hydrophobic interactions in G103C-T113C increased, which are possibly other factors contributing to the change, respectively. Throughout the studies on molecular modification of alginate lyases published in recent years, rational design is a common method, especially the introduction of disulfide bonds [88]. Even though it is an effective strategy, it still needs to be based on an analysis of the enzyme structure. At the same time, it is necessary to consider more closely the selection of valid appropriate disulfide bonds that are predicted by computational tools; otherwise, the activity will not change or even decrease [86].

### 2.8. Applications of Alginate Lyases

Even though alginate lyases have many advantages in industrial applications, most of them are still affected by some factors, which limited their commercial application. One of the commercially available alginate lyases, FlAlyA, was released on the market as “HULK alginate lyase” by Nippon Gene (Tokyo, Japan) in 2015, and it has been used to identify the species of brown algae. Due to the similarities between nucleic acids and alginates, it was difficult to distinguish them and quickly purify the nucleic acids using commercial extraction methods from brown algae. However, using HULK alginate lyase can efficiently degrade alginates, which eliminates interference and help to quickly extract high-quality DNA and RNA commercially [89].

#### 2.8.1. Antibiotic Applications

Dense colonization of mucoid *Pseudomonas aeruginosa* within the self-secreted extracellular matrix, called biofilm, is a principal reason for the failure of antimicrobial therapy in cystic fibrotic patients [90]. As a protective barrier, the thick biofilm is composed of several components [91]. Alginates are the major component of *P. aeruginosa* biofilm, which is responsible for surface adhesion and stabilization of biofilm to resist antibiotics [92,93]. Moreover, alginates can interrupt the phagocytosis of macrophages and neutrophils, limit the lymphocyte function, and induce inflammatory reactions to aggravate the lung infection [91]. Although various factors contribute to a successful infection in the lungs of cystic fibrosis patients, alginate has been one of the best-studied key indicators of chronic lung infection. Since normal human cells do not contain alginate, alginate lyase is a good choice for breaking up the biofilms of *P. aeruginosa* in the human body for less toxicity. In view of that, many studies have reported the application of alginate lyases in treating the infection. Núria et al. observed the contradiction between different reports, and they tested five different alginate lyases in order to find the secrets behind the contradiction. According to their results, only enzymes with polyM/G activity are effective in dissolving biofilms and have a synergistic effect with ciprofloxacin antibiotic, while strict polyM or polyG specific alginate lyases show fewer activities and do not have the synergistic effect [92], which verifies that substrate specificity acts as a key factor in pharmaceutical applications, as described above. Here, this paper summarizes some online studies posted in recent years.

Bifunctional alginate lyase degrading both polyM and polyG could prevent growth and eradication of *P. aeruginosa* sp. TAG48 biofilm and exhibits synergy with tobramycin and cefixime but not with ciprofloxacin [93]. The results indicate that the use of purified novel alginate lyase with antibiotics could be a beneficial alternative for the treatment of *P. aeruginosa* infections.

Mahajan et al. [94] assessed the inhibitory effects of seven lyases on biofilm formation. They used PslG, a promising candidate for disrupting the biofilm of *P. aeruginosa*, as a comparison. Among them, four kinds of alginate lyases—CaAly (endolytic lyase), VspAlyVI (endolytic lyase), FspAlyFRB (exolytic lyase), and SA1-IV (exolytic lyase)—inhibited biofilm formation of *P. aeruginosa* strains isolated from the sputum of a cystic fibrosis patient. Although their results demonstrated several alginate lyases with the potential to restrain the formation of biofilms of *P. aeruginosa* in cystic fibrosis, they could not demonstrate alginate degradation upon incubation of alginate lyases with sputum. Further in vivo experiments are needed in future studies.

In some research studies, alginate lyases have been combined with drug delivery systems to enhance the effect of antibiotics, targeting alginate [91]. In one study, a silver nanocomposite composed of silver nanoparticles and a mesoporous organosilica layer was created to deliver two pharmaceutical compounds (alginate lyase and ceftazidime) to digest the alginates that help ceftazidime to kill *P. aeruginosa* from the lungs. Further, they carried out in vivo experiments, in which they used silver nanoparticles to treat the infected mouse, and their results showed the silver nanoparticles they designed successfully eradicated the established *P. aeruginosa* PAO1 from the mouse lungs and relieved the lung injuries. For the effective treatment of *P. aeruginosa* infection, alginate lyase functionalized chitosan nanoparticles of ciprofloxacin were developed and exhibited significantly higher inhibitory effect in cystic fibrosis patients and reduced the biomass, thickness, and density in vitro without any toxicity [90]. The result suggested the proposed strategy to be a better alternative for the effective treatment of cystic fibrosis infections.

Attention should be directed to the fact that growth patterns of clinical isolates in vitro may differ from those in vivo with unpredictable patterns of biofilm development; therefore, the use of bioactive compounds in clinical studies should benefit from laboratory methods improved in vitro and in vivo, which should be discussed further.

#### 2.8.2. Preparation of Alginate Oligosaccharides

AOs prepared by enzymatic methods show some special bioactivities by reason of the unsaturated bond at the reducing end [95,96]. AOs have potentials such as antitumor [97], antidiabetic [98], antihypertensive [99], anti-inflammatory [100,101], antimicrobial [102], antioxidant [103], anticancer [104], immunomodulatory [3,105] and anti-radiation [76,106] properties. The bioactivities of alginate oligosaccharides are closely related to their DPs and structures [5,107], so DPs of oligosaccharides produced by endolytic alginate lyase have been of interest.

Previous studies were mostly conducted on the laboratory level to discover new Alginate lyases that can produce different AOs, which showed that products of endolytic alginate lyase own DPs generally ranging from DP2 to DP5 [107]. For example, a salt-activated enzyme AlgSH7 from PL7 can produce oligosaccharides ranging from DP2 to DP4 using alginate and polyM as substrates [56]. Another polyG-preferred alginate lyase, AlyF, regards oligosaccharides with DP3 as the dominant product [38]. An alginate lyase from *Isoptericola halotolerans*, CGMCC 5336, can only perform elimination on guluronic acid residue (act on G m or G-G) and produce oligo-fractions with DP2-4, which were separated by Bio-Gel P2 column and assayed by TLC and ESI-MS [108].

Undoubtedly, DPs of alginate oligosaccharides are related to the degradation process under different conditions. For example, alginate lyase BcelPL6 firstly created DP5, which would be further degraded into DP2 and DP3 using polyM as substrates; however, the process changed when substrate turned to alginate: AOs with DP2-7 were produced in the initial stage, and DP4 and DP6 increased as time passed, and finally, the DP2 became the main component in the end products [14]. Another alginate lyase AlyPL6, a novel member of PL6 with high activity, shows more preference toward polyMG than other poly blocks and regards tetrasaccharide as the minimal substrate [109]. At the initial stage of the reaction, various oligosaccharides with different DPs are produced, which will be further degraded with the increase in time. At the final stage of the reaction, oligosaccharides with DP1-4 are detected. It can be derived from previous studies that most of the products of alginate lyases are not single, which is also one of the problems limiting their application. Since specific DPs are the key factors for the function of alginate oligosaccharides, how to control the purity of the products and how to quickly separate the pure products more economically are worthy of attention.

Additionally, some endolytic lyases can cooperate with exolytic lyases, resulting in a strong synergistic effect on alginate degradation. When endolytic lyase AlyPB1 and exolytic lyase AlyPB2 cooperated together, the conversion rate of alginate polysaccharides to unsaturated monosaccharides was significantly increased—approximately sevenfold than when AlyPB2 was used alone [24]. In view of the strong benefits of synergistic effect, it is believed that synergistic degradation should be one of the research priorities.

#### 2.8.3. Preparation of Pharmaceutical Intermediate

Pyruvate is an essential pharmaceutical intermediate, which has been widely used as a starting material in the biosynthesis of pharmaceuticals, such as L-tryptophan, L-tyrosine, alanine, and L-DOPA [110]. Most of the reported studies produce pyruvate from glucose, and studies in the literature regarding the production of pyruvate from other sources are rare, especially alginate [111]. During the process of generating pyruvate, DEH produced by exolytic alginate lyases can be reduced to 2-keto-3-deoxy-D-gluconate (KDG) which enters the Entner−Doudoroff pathway to produce two molecules of pyruvate [112]. An LDH knockout, *Sphingomonas* sp. strain A1, can secrete pyruvate from alginates using endolytic and exolytic alginate lyases at high aeration rates [113]. In the presence of 5% (*w*/*v*) initial alginate concentrations, the maximum pyruvate concentration and productivity of pyruvate were 4.56 g/L and 95.0 mg/L/h, which indicate alginate is a promising C-source for bioproduction of pyruvate. These basic data provided are beneficial for biorefinery platform of brown algae involved in alginate lyases to produce pharmaceutical materials.

## 3. Conclusions

Alginate lyases attract worldwide attention for their unique characteristics, which render great importance for their potential in pharmaceutical applications. Although there have been substantial studies on alginate lyase genes, especially from marine bacteria, there is an immediate need to clone alginate lyase genes from other organisms and understand their characteristics. Moreover, the reported exolytic alginate lyases are relatively less than endolytic alginate lyases, which indicated there is considerable scope for screening novel exolytic alginate lyases. Moreover, the PL families that include alginate lyases have expanded to 14 families, and some newly discovered alginate lyases exhibit novel structure and action mechanisms. However, structure information is still not obtained enough to explain every mechanism of the fourteen families.

Currently, pharmacological applications of alginate lyases are well documented, though the molecular biology part of alginate lyases is not extensively explored. More and more alginate lyases with special properties are discovered, which need to be further characterized for biopharmaceutical production. For example, kinetic analysis of an enzyme is an important factor to find its utility at the commercial level but unfortunately, most of the cold-adapted alginate lyases reported so far lack detailed kinetic analysis. On the other hand, the knowledge of the factors or the regulatory elements involved in the expression of alginate lyase genes, especially exolytic and cold-adapted lyase genes, could be beneficial for molecular modification and thus increase the catalytic efficiency, special properties, and yield to meet the commercial requirements for its application in the pharmacological industry. Therefore, there is a scientific need to study the molecular biology of alginate lyases so that a large amount of these enzymes could be produced by their overexpression and pharmaceutical application.

## Figures and Tables

**Table 1:** Structural characters of typical alginate lyases that have been characterized.

Name	PL Family	Structure	EC	PDB	Refence
AlgL	PL5	(*α*/*α*)_n_ toroid fold	4.2.2.3	4OZV	[40]
A1-III	PL5	(*α*/*α*)_n_ toroid fold	4.2.2.3	1HV6	[39]
BcelPL6	PL6	*β*-helix	4.2.2.3	6QPS	[14]
AlyGC	PL6	*β*-helix	4.2.2.11	5GKD	[29]
AlyF	PL6	*β*-helix	4.2.2.11	5Z9T	[38]
AlyPG	PL7	*β*-jelly roll	4.2.2.11	1UAI	[43]
AlgAT5	PL7	*β*-jelly roll	NA	5ZQI	NA
FlAlyA	PL7	*β*-jelly roll	4.2.2.3	5Y33	[44]
AlyA	PL7	*β*-jelly roll	4.2.2.11	4OZX	NA
AlyQ	PL7	*β*-jelly roll	4.2.2.3	5XNR	[45]
PA1167	PL7	*β*-jelly roll	4.2.2.-	1VAV	[46]
AlyC3	PL7	*β*-jelly roll	4.2.2.3	7C8G	[47]
A1-II’	PL7	*β*-jelly roll	4.2.2.11	2CWS	[48]
AlyA5	PL7	*β*-jelly roll	4.2.2.26	4BE3	[49]
AlyA1	PL7	*β*-jelly roll	4.2.2.11	3ZPY	[49]
Psalg7A	PL7	*β*-jelly roll	4.2.2.3	6YWF	NA
PsMan8A	PL8	NA	NA	NA	[50]
vAL-1	PL14	*β*-jelly roll	4.2.2.14	3A0N	[51]
AkAly30	PL14	*β*-jelly roll	4.2.2.3	5GMT	[52]
Atu3025	PL15	(*α*/*α*)_n_ toroid fold + anti-parallel *β*-sheet	4.2.2.26	3A0O	[35]
AlyA3	PL17	(*α*/*α*)_n_ toroid fold + anti-parallel *β*-sheet	4.2.2.3	7BJT	[42]
Alg17c	PL17	(*α*/*α*)_n_ toroid fold + anti-parallel*β*-sheet	4.2.2.26	4OK2	[36]
Aly-SJ02	PL18	*β*-jelly roll	4.2.2.-	4Q8K	[53]
P84143	PL18	*β*-jelly roll	4.2.2.-	1J1T	NA
PsAly	PL31	*β*-helix	4.2.2.3	6KFN	[54]
Aly36B	PL36	*β*-jelly roll	4.2.2.3	6KCV	[30]
Dp0100	PL39	(*α*/*α*)_n_ toroid fold + anti-parallel*β*-sheet	4.2.2.-	6JP4	[41]

**Table 2:** Alginate lyases that can be activated by NaCl. Their activities can be increased multiple times at the appropriate concentration of NaCl.

Name	PL	Source	Optimal Concentration of NaCl	Optimal Enzymatic Condition	Km	Vmax	kcat	Enzyme Activity ^a^	Reference
AlgM4	PL7	Marine bacterium *Vibrio weizhoudaoensis M0101*	1 mol/L	30 °C; pH = 8.5	Km = 2.72 mg/mL for sodium alginate	Vmax = 2.75 nmol/s for sodium alginate	kcat = 30.25 S^−1^ for sodium alginate	7-fold increase	[61]
AlyPM	PL7	Marine bacterium *Pseudoalteromonas* sp. SM0524	0.5–1.2 mol/L	30 °C; pH = 8.5	Km = 3.15 mg/mL (in 0.5 M NaCl) for sodium alginate Km = 74.39 mg/mL (in 0 M NaCl) for sodium alginate	NA	NA	6-fold increase	[58]
rA9mT	PL7	Deep-sea bacterium *Vibrio* sp. JAM-A9m	0.4 mol/L	30 °C in the presence of 0.2 M NaCl at pH 7.5	NA	NA	NA	24-fold increase	[62]
A1m	PL7	*Agarivorans* sp. JAM-A1m from a deep-sea sediment	0.6–0.8 mol/L	30 °C either in the presence of 0.2 M NaCl at pH 9 or in its absence at pH 10	NA	Vmax values are 38.4, 285.7, 416.7, and 526.3 units mg^−1^ protein in the presence of 0, 0.1, 0.2, and 0.5 M NaCl, respectively for sodium alginate	NA	20-fold increase	[63]
AlyC3	PL7	*Psychromonas* sp. C-3 isolated from the Arctic brown alga *Laminaria*	0.5 mol/L	20 °C; pH = 8.0	Km = 0.24 ± 0.05 mg/mL, for polyM	Vmax = 19,704.73 ± 1865.49 U/mg for polyM	NA	2.9-fold increase	[47]
Aly08	PL7	Marine bacterium *Vibrio* sp. SY01	0.3 mol/L	45 °C; pH = 8.35	NA	NA	NA	8-fold increase	[64]

^a^ The increase multiple of enzyme activity in the presence of appropriate concentration of NaCl.

**Table 3:** Alginate lyases show optimal activity at alkaline conditions and keep relatively stable under a wide pH range.

Name	Origin/Strain	PL	Optimal pH	Relative Activity at Various pH Values	Km	Vmax	kcat	Reference
Alyw202	*Vibrio* sp. W2	PL7	9.0	>80% ^a^ (pH 5.0–9.0) >60% ^a^ (pH 3.0–10.0)	NA	NA	NA	[66]
Aly08	*Vibrio* sp. SY01	PL7	8.35	>80% ^a^ (pH 4.0–10.0) >60% ^a^ (pH 7.0–11.0)	NA	NA	NA	[64]
AlgNJ–04	*Vibrio* sp. NJ04	PL7	7.0	>60% ^b^ (pH 4.0–10.0)	Km = 0.49, 0.86, 0.24 mM, respectively, for alginate, polyM and polyG	Vmax = 72, 95, 35 pmol/s, respectively, for alginate, polyM and polyG	kcat = 59, 77, 29 s^−1^, respectively, for alginate, polyM and polyG	[65]
AlgH	*Marinimicrobium* sp. H1	PL7	10.0	>60% ^a^ (pH 6.0–10.0)	Km = 6.6 ± 2.2, 7.6 ± 1.6, 9.1 ± 2.4 mg·mL^−1^, respectively, for sodium alginate, polyG and polyM	Vmax = 224.6 ± 33.6, 146.6 ± 15.6, 62.6 ± 8.8 U·mg of protein^−1^, respectively, for sodium alginate, polyG and polyM	kcat = 260.6 ± 36.2, 155.7 ± 17.1, 66.8 ± 6.7 s^−1^, respectively, for sodium alginate, polyG and polyM	[67]
Alg823	*Pseudoalteromonas carrageenovora* ASY5	PL6	8.0	>80% ^b^ (pH 6.0–10.0)	Km = 0.15 mg/mL for sodium alginate	Vmax = 1.84 U/g for sodium alginate	1.19 × 10^6^ s^−1^ for sodium alginate	[16]
AlgNJ–07	*Serratia marcescens* NJ–07	NA	9.0	>80% ^c^ (pH 7.0–10.0)	Km = 0.53, 0.27 mM, respectively, for sodium alginate and polyM	Vmax = 74, 67 nmol/s, respectively, for sodium alginate and polyM	kcat = 34, 31 s^−1^, respectively, for sodium alginate and polyM	[68]

^a^ Incubating at 4 °C for 12 h; ^b^ Incubating at 4 °C for 24 h; ^c^ Incubating at 40 °C for 24 h.

**Table 4:** Alginate lyases with good thermal stability.

Name	Origin	PL	Optimal Temperature	Thermal Stability	Km	Vmax	kcat	Reference
rSAGL	*Flavobacterium* sp. H63	NA	45 °C	Retained 49% of activity at 50 °C for 72 h	Km = 4.63 mg/mL (from *P. pastoris*), 4.64 mg/mL (from *E. coli*) for sodium alginate	NA	NA	[71]
rNitAly	*Nitratiruptor* sp. SB155-2	PL7	70 °C	Retained 50% of activity at 67 °C for 30 min	NA	NA	NA	[74]
Alg823	*Pseudoalteromonas carrageenovora* ASY5	PL6	55 °C	Retained over 75% of the maximum activity at 50 °C for 30 min	Km = 0.15 mg/mL for sodium alginate	Vmax = 1.84 U/g for sodium alginate	1.19 × 10^6^ s^−1^ for sodium alginate	[16]
AlgC-PL7	*Cobetia* sp. NAP1	PL7	45 °C	Retained 60 and 30% of activity at 80 and 90 °C for 1 h	NA	NA	NA	[75]
AMOR_PL17A	Arctic Mid-Ocean Ridge (AMOR) metagenomics data set	PL17	High temperature (>50 °C)	Retained 100% of activity at 60 °C for 24h (in the absence of substrate)	NA	NA	NA	[73]
ALW1	*Microbulbifer* sp. ALW1	NA	45 °C	Retained 68% of activity at 45 °C for 1 h	Km = 1.03 mg/mL for sodium alginate	Vmax = 4.63 U/mg for sodium alginate	kcat = 69.38 s^−1^ for sodium alginate	[76]

**Table 5:** Summary of alginate lyases with cold-adapted properties.

Name	PL	Source	Substrate Preference	Action Mode	Optimal Temperature	Cold-Adapted Property	Km	Vmax	kcat	Main Products	Reference
AlyC3	PL7	*Psychromonas* sp. C-3 isolated from the Arctic brown alga *Laminaria*	polyM	Endolytic lyase	20 °C	Retained 48.2% of maximum activity at 1 °C; Unstable at 30 °Cand above	Km = 0.24 ± 0.05 mg/mL, polyM	Vmax = 19,704.73 ± 1865.49 U/mg for polyM	NA	ΔMM (Δ represents 4-deoxy-L-*erythro*-hex-4- enopyranosyluronic acid)	[47]
AlgSH7	PL7	*Microbulbifer* sp. SH-1	polyM	Endolytic lyase	40 °C	Retained 80% of the maximum activity over at 25 °C; Unstable at temperatures beyond 30 °C	NA	NA	NA	Disaccharides, tri-saccharides, and tetrasaccharides	[56]
TsAly6A	PL6	*Thalassomonas* sp. LD5	polyG	Endolytic lyase	35 °C	Retained 73.1% and 21.1% of the maximum activity at 20 °C and 10 °C; Retained over 90% of initial activity after incubation at 30 °C for 1 h, but only 29% remained at 40 °C	NA	NA	NA	Disaccharides and trisaccharides	[70]
Alys1	PL7	*Tamlana* sp. s12	polyM	Exolytic lyase	35 °C	Activity reduced at temperatures above 35 °C; Retained more than 50% of the maximum activity at 10 °C; No detectable activity at 60 °C	Km = 0.20 ± 0.01 mM for sodium alginate	NA	kcat = 4.43 ± 0.027 s^−1^ for sodium alginate	Monosaccharides, disaccharides, trisaccharides and some lower monomers	[78]
AlyS02	PL7	*Flavobacterium* sp. S02	bifunctional	Endolytic lyase	30 °C	Retained more than 70% of relative activity in the range of 20–40 °C (retained more than 90% of maximum activity at 25 °C); Activity reduced at temperatures above 40 °C	NA	NA	NA	Disaccharides and trisaccharides	[79]
AlgM4	PL7	*Vibrio weizhoudaoensis* M0101	bifunctional	Endolytic lyase	30 °C	Retained 92% of its initial activity after a 30 min incubation at 30 or 35 °C (in the presence of 1 mol/L NaCl); Decreased rapidly at temperatures exceeding 40 °C and decreased by 63% at 45 °C	Km = 2.72 mg/mL , for sodium alginate	Vmax = 2.75 nmol/s for sodium alginate	kcat = 30.25 S^−1^ for sodium alginate	Oligosaccharides with DP of 2–9	[61]
Alg2951	PL7	*Alteromonas portus* HB161718^T^	polyG	Endolytic and exolytic lyase	25 °C	Retained over 60% of the maximum activity at the temperature range of 15–40 °C; No detectable activity at 60 °C	NA	NA	NA	Monosaccharides and trisaccharides	[80]
AlyL1	PL7	*Agarivorans* sp. L11	bifunctional	Endolytic lyase	40 °C	Retained 54.5% and 72.1% of the maximum activity at 15 °C and 20 °C	NA	NA	NA	Disaccharides and trisaccharides	[81]
AlyPM	PL7	*Pseudoalteromonas* sp. SM0524	polyM	Endolytic lyase	30 °C	Retained 19% of the maximum activity at 5 °C; No detectable activity at 45 °C	Km = 3.15 mg/ml (in 0.5 M NaCl) for sodium alginate Km = 74.39 mg/ml (in 0 M NaCl) for sodium alginate	NA	NA	Oligosaccharides with DP of 2–3	[58]
Alyw201	PL7	*Vibrio* sp. W2	polyG	Endolytic lyase	30 °C	Retained more than 80% activity at 25–40 °C; Retained 72.9% and 38.4% of the highest activity at 10 °C and 20 °C	NA	NA	NA	Oligosaccharides with DP of 2–6	[77]
ZH0-IV	NA	*Sphingomonas* sp. ZH0	bifunctional	Exolytic lyase	37 °C	Retained more than 80% of activity after incubating in phosphate buffer with an optimum pH for 10 min between 25 and 42 °C	Km = 0.41 mg/ml for sodium alginate	Vmax = 5.53 U/ml for sodium alginate	NA	Monosaccharides	[82]
AlyGC	PL6	*Glaciecola chathamensis* S18K6^T^	polyG	Exolytic lyase	30 °C	Activity rapidly decreased above 30 °C; Retained relatively high activity between 10 and 30 °C	NA	NA	NA	Monosaccharides	[29]
AlgNJU-03	PL7	*Vibrio* sp. NJU-03	bifunctional	Endolytic lyase	30 °C	Retained approximately 40% activity after incubation at 40 °C for 30 min and was gradually inactivated as temperature increased	Km = 8.50, 10.94, 4.00 mM, respectively for sodium alginate, polyM and polyG	Vmax = 1.67, 0.30, 2.50 nmol/s, respectively for sodium alginate, polyM and polyG	kcat = 30.64, 5.50, 45.87 s^−1^, respectively for sodium alginate, polyM and polyG	Disaccharides, trisaccharides and tetrasaccharides	[83]
rA9mT	PL7	*Vibrio* sp. JAM-A9m	polyM	NA	30 °C	Relative activities at 10 °C and 2 °C were around 45% and 30% of the maximal activity; enzyme was rapidly inactivated after incubating at above 40 °C in the absence of NaCl.	NA	NA	NA	NA	[62]
TsAly7B	PL7	*Thalassomonas* sp. LD5	bifunctional	Endolytic lyase	30 °C	Retained approximately 60% of relative activity at 20 °C; Unstable at temperatures beyond 30 °C	NA	NA	NA	Disaccharides, trisaccharides, and tetrasaccharides	[84]

## Data Availability

Not applicable.
